# Impact of gut microbiota on human immune system development: A longitudinal study

**DOI:** 10.6026/973206300215015

**Published:** 2025-12-15

**Authors:** Dipalben Shreyaskumar Patel, Mukul Singh, Vivekanand Ashok, Amrit Podder, Jorge Antonio Segovia Guerrero, Renato Matías Torres Montano, Abhishek Kumar Semwal, Luis Patricio Dávila Aguilar, Karina Vanessa Herrera Riofrío

**Affiliations:** 1 Department of Obstetrics and Gynecology, Akshaydeep Hospital, Gujarat, India; 2 Department of General Surgery, All India Institute of Medical Sciences (AIIMS), Gorakhpur, Uttar Pradesh, India; 3 Department of ENT - Head and Neck Surgery, Azeezia Health City, Palakkad, Kerala, India; 4 Department of Physiology, Teerthanker Mahaveer Medical College & Research Centre, Teerthanker Mahaveer University, Moradabad, Uttar Pradesh, India; 5 Department of Emergency, Armed Forces Specialty Hospital No. 1, Quito, Ecuador; 6 Teaching Analyst, Armed Forces Specialty Hospital No. 1, Quito, Ecuador; 7 Kayachikitsa, Ankerite Ayurvedic Medical College and Hospital, Lucknow, India; 8 Luis Patricio Dávila Aguilar, Internal Medicine Physician - Hematology, Armed Forces Specialties Hospital No. 1, Quito, Ecuador; 9 Department of Nutrition and Dietetics Specialty, Leader of the Nutrition and Dietetics Specialty at HE-1, Armed Forces Specialty Hospital No. 1, Quito, Ecuador

**Keywords:** Antibiotic use, diversity, guts microbiota, immune markers, longitudinal study

## Abstract

The development of the immune system in early childhood is influenced by various factors, and the role of gut microbiota in this process remains a critical area of investigation. Therefore, it is of interest to investigate the impact of gut microbiota on immune system development in 110 children over three years. It found significant correlations between microbiota diversity and immune markers, with higher diversity linked to improved immune regulation. The study also highlighted the negative effects of early antibiotic use and the positive influence of a fiber-rich diet on gut microbiota composition and immune function. Thus, we show the critical role of early- life microbial exposures in shaping immune health.

## Background:

The human immune system is a complex and intricate network that is crucial in defending the body against harmful pathogens [1]. This system is composed of various components, such as white blood cells, antibodies and lymphatic organs, all of which work together to detect and eliminate foreign invaders [2]. However, the development and functioning of this immune system are not solely dependent on genetic factors. A growing body of research has shown that environmental factors, particularly the gut microbiota, play a significant role in shaping immune responses and promoting overall immune health [3]. Gut microbiota refers to the vast and diverse community of microorganisms, including bacteria, fungi, viruses and other microbes, that inhabit the human gastrointestinal tract [4]. These microorganisms are not mere passive residents; rather, they actively participate in several bodily functions, including digestion, metabolism and the regulation of immune responses. The relationship between gut microbiota and the immune system is a dynamic and evolving one [5]. Early-life exposure to various microbial communities is thought to be critical for the development of a functional and balanced immune system. It is during infancy and early childhood that the immune system undergoes substantial maturation and the gut microbiota plays a pivotal role in this process [6]. Research has shown that the diversity and composition of the gut microbiota can significantly influence the development of the immune system [7]. For instance, a healthy and balanced microbiome can enhance the immune system's ability to distinguish between harmful pathogens and benign substances, while an imbalanced or altered microbiome may contribute to immune system dysfunction, leading to the development of allergies, autoimmune diseases and other immunological disorders [8]. Furthermore, certain strains of bacteria found in the gut are known to interact with the immune system, promoting the production of specific immune cells and the secretion of various cytokines that help regulate immune responses [9]. The timing and nature of microbial exposure are key factors in how the gut microbiota influences immune system development. Studies have indicated that disruptions in early-life microbial exposure, such as those caused by the overuse of antibiotics, cesarean section births, or lack of breastfeeding, may result in an increased risk of immune-related diseases [10]. This highlights the importance of understanding how the gut microbiota interacts with the immune system, particularly during critical windows of development, such as infancy and early childhood [11]. Despite the growing interest in this area, much remains unknown about the precise mechanisms through which gut microbiota influences immune development. Longitudinal studies, which track individuals over extended periods of time, are particularly valuable in providing insights into the long-term effects of gut microbiota on immune system development [12]. By observing changes in the microbiota and immune function over time, researchers can better understand how early-life microbial exposures impact immune health later in life [13]. Therefore, it is of interest to describe because it seeks to explore the long-term effects of gut microbiota on the development and regulation of the human immune system.

## Methodology:

This study employed a longitudinal research design to examine the impact of gut microbiota on the development of the human immune system. A total of 110 participants were recruited for this study. The participants were selected from a cohort of children, with careful attention given to including a diverse sample in terms of age, sex and health status. The study spanned several years to track changes in the gut microbiota composition and immune system development over time. The participants were children aged between 6 months and 2 years at the start of the study. This age range was chosen because early childhood is a critical period for immune system development and microbial exposure. Participants were recruited from local pediatric clinics, ensuring a diverse and representative sample of the population. Parents or guardians provided written informed consent for their child's participation and the study was approved by the relevant ethical review board.

## Inclusion criteria included:

[1] Healthy children without any known chronic diseases or conditions that might influence immune function (e.g., autoimmune disorders, chronic infections).

[2] Children who had not received immunosuppressive therapy or antibiotics in the last three months prior to the study.

## Exclusion criteria included:

[1] Children with a history of gastrointestinal disorders that might affect gut microbiota.

[2] Children who had received a course of antibiotics or immunosuppressive treatments within the past three months.

The sample size of 110 participants was determined based on statistical power analysis, which suggested that this number would provide sufficient power to detect significant differences in the microbiota and immune responses over the course of the study. This sample size allowed for meaningful subgroup analyses and ensured that the study could detect small to moderate effects with adequate confidence. Data were collected at multiple time points during the study. Initial baseline data were gathered at the start of the study and follow-up data were collected every six months for a period of three years.

## The primary data collection methods included:

Stool samples were collected from each participant at each follow-up point to assess the composition and diversity of their gut microbiota. These samples were analyzed using 16S ribosomal RNA (rRNA) sequencing to identify the different bacterial communities present in the gut. DNA extraction and sequencing were performed at a certified laboratory specializing in microbiome analysis. Blood samples were taken at each follow-up to measure various immune markers, including levels of cytokines, antibodies and immune cell counts. These biomarkers provided insights into the state of immune function at each time point. Specific focus was placed on measuring T-cell responses, B-cell function and other immune system indicators that reflected the development of adaptive and innate immunity. Parents completed questionnaires regarding their child's diet, birth history (e.g., mode of delivery, breastfeeding), antibiotic use and any other relevant health information. This helped identify potential confounding factors that might influence both microbiota composition and immune development. Descriptive statistics were used to summarize the demographic characteristics of the study population. Longitudinal data analysis techniques, such as mixed-effects models, were applied to assess changes in gut microbiota diversity and immune markers over time. The relationship between microbiota composition and immune system development was examined using correlation analyses and regression models, adjusting for potential confounding variables such as diet, birth mode and antibiotic exposure. Additionally, subgroup analyses were performed to explore potential differences in the impact of gut microbiota on immune development across various participant groups, including those with different dietary habits or early-life exposures. The study was conducted in compliance with ethical guidelines for research involving children. Confidentiality was maintained throughout the study and all data were anonymized. Participants and their families were informed about the study's purpose, procedures and any potential risks and informed consent was obtained before participation. Parents or guardians had the right to withdraw from the study at any time without penalty. In summary, this study aimed to provide a comprehensive analysis of how gut microbiota influenced immune system development by following a cohort of 110 children over several years. The combination of microbiota analysis, immune system assessments and health history data allowed for a detailed understanding of these complex interactions and their long-term effects on health.

## Results:

The study assessed the impact of gut microbiota on the development of the immune system over three years. The data collected included the composition of gut microbiota, immune markers and demographic factors, such as diet and antibiotic use. The results showed significant correlations between gut microbiota composition and immune system development. These findings were consistent across the follow-up periods, with notable changes in immune markers and microbiota composition observed as participants aged. Table 1 (see PDF) presents the diversity of the gut microbiota in participants at baseline and after each follow-up period. The results indicated a significant increase in microbiota diversity over time, with the greatest changes observed between the first and second years of life. The increase in diversity was accompanied by shifts in the relative abundance of specific bacterial families, with a notable rise in beneficial Firmicutes and Bacteroidetes and a decrease in potentially harmful Proteobacteria. The microbiota showed an overall increases in diversity over the study period, which correlated with an improvement in immune markers, particularly the levels of T-cells and cytokines. Table 2 (see PDF) presents the changes in immune markers over time. The levels of pro-inflammatory cytokines such as TNF-α and IL-6 were found to decrease significantly over the course of the study, while regulatory cytokines like IL-10 increased. These shifts were most prominent in participants who had a balanced microbiota, as indicated by the diversity indices in Table 1 (see PDF). The increase in IL-10 and T-cell counts suggests that a more diverse gut microbiota was associated with improved regulation of the immune system, potentially reducing the risk of inflammation-related diseases. Table 3 (see PDF) highlights the impact of antibiotic use during early childhood on gut microbiota composition and immune system development. Participants who had received antibiotics during the first year of life exhibited reduced microbiota diversity and a higher proportion of potentially harmful bacteria, such as Proteobacteria. These participants also had lower T-cell counts and higher levels of pro-inflammatory cytokines. These findings suggest that early antibiotic use could negatively affect immune system development by altering the gut microbiota composition, potentially leading to less effective immune regulation. Table 4 (see PDF) presents the effects of diet on gut microbiota composition. Participants who consumed a diet rich in fiber, fruits and vegetables had a higher diversity of gut microbiota, as well as a greater abundance of beneficial bacteria such as Firmicutes and Bacteroidetes. This group also had higher levels of regulatory immune markers such as IL-10. These results demonstrate that diet plays a critical role in shaping the gut microbiota and, consequently, in influencing immune system development. A diet high in fiber and plant- based foods supports a healthier gut microbiome, which in turn enhances immune regulation. Overall, the results indicated that gut microbiota diversity and composition were strongly correlated with immune system development over the study period. Participants with higher microbiota diversity, especially those who had minimal antibiotic use and consumed a diet rich in fiber, exhibited improved immune system function. These individuals showed lower levels of pro-inflammatory cytokines and higher levels of regulatory cytokines, suggesting that a balanced gut microbiota is beneficial for immune regulation. The results from this study underline the importance of early microbial exposures and lifestyle factors, such as diet, in shaping immune system development and suggest that interventions aimed at promoting a healthy gut microbiota could be beneficial for long-term immune health.

## Discussion:

This longitudinal study investigated the relationship between gut microbiota composition and immune system development in children over a three-year period. Our findings revealed significant associations between microbiota diversity and immune markers, consistent with previous research. The increase in gut microbiota diversity observed in our study aligns with findings from a study by Wernroth *et al.* (2022) [14], which reported a convergence toward a more diverse, adult-like microbiota composition in children over the first two years of life. Similarly, Tsuruoka *et al.* (2024) [15] found that the alpha diversity of the gut microbiota in children increased with age, approaching adult levels by 3.5 years. Our study also noted a decrease in pro-inflammatory cytokines and an increase in regulatory cytokines over time, particularly in participants with higher microbiota diversity. This observation supports the findings of Dera *et al.* (2025) [16], who highlighted the role of early-life microbiota in regulating immune responses and preventing inflammatory diseases. The role of diet in shaping the gut microbiota and influencing immune function, as demonstrated in our study, is supported by the work of Aziz *et al.* (2024) m, who discussed the significant changes in gut microbiota composition during the first year of life and its interactions with the developing immune system[17].

## Conclusion:

The critical role of gut microbiota in immune system development during early childhood is shown. The observed associations underscore the importance of maintaining a balanced microbiota through factors such as minimal antibiotic use and a diet rich in fiber to support optimal immune function.
